# Effect of Fidarestat and α-Lipoic Acid on Diabetes-Induced Epineurial Arteriole Vascular Dysfunction

**DOI:** 10.1080/15438600490277824

**Published:** 2004

**Authors:** M. A. Yorek, L. J. Coppey, J. S. Gellett, E. P. Davidson, D. D. Lund

**Affiliations:** 1 Veterans Affairs Medical Center and Department of Internal Medicine University of Iowa Iowa City Iowa 52246 USA

## Abstract

In the present study, the authors examined whether treating streptozotocin-induced diabetic rats with the combination of *α*-lipoic acid and fidarestat, an aldose reductase inhibitor, can promote the formation of dihydrolipoic acid in diabetic animals and thereby enhance the efficacy of *α*-lipoic acid as monotherapy toward preventing diabetic vascular and neural dysfunction.Treating diabetic rats with the combination of 0.25% *α*-lipoic acid (in the diet) and fidarestat (3 mg/kg body weight) prevented the diabetesinduced slowing of motor nerve conduction velocity and endoneurial blood flow. This therapy also significantly improved acetylcholine-mediated vasodilation in epineurial arterioles of the sciatic nerve compared to nontreated diabetic rats. Treating diabetic rats with 0.25% *α*-lipoic acid and fidarestat (3 mg/kg body weight) was equally or more effective in preventing vascular and neural dysfunction than was monotherapy of diabetic rats with higher doses of *α*-lipoic acid or fidarestat. Treating diabetic rats with the combination of 0.25% *α*-lipoic acid and fidarestat (3 mg/kg body weight) significantly improved several markers of oxidative stress and increased the serum levels of both *α*-lipoic acid and dihydrolipoic acid. These studies suggest that combination therapy consisting of *α*-lipoic acid and fidarestat may be more efficacious in preventing diabetes-induced vascular and neural dysfunction in peripheral tissue compared to monotherapy, which requires higher doses to be equally effective. The effect of this combination therapy may in part be due to the increased production and/or level of dihydrolipoic acid.


*Experimental Diab. Res.*, 5:123–135, 2004
Copyright © Taylor and Francis Inc.
ISSN: 1543-8600 print / 1543-8619 online
DOI: 10.1080/15438600490277824

### Effect of Fidarestat and $\alpha$-Lipoic Acid on Diabetes-Induced Epineurial Arteriole Vascular Dysfunction

**M. A. Yorek, L. J. Coppey, J. S. Gellett, E. P. Davidson, and D. D. Lund**

*Veterans Affairs Medical Center and Department of Internal Medicine, University of Iowa, Iowa City, Iowa, USA*

**In the present study, the authors examined whether treating streptozotocin-induced diabetic rats with the combination of $\alpha$-lipoic acid and fidarestat, an aldose reductase inhibitor, can promote the formation of dihydrolipoic acid in diabetic animals and thereby enhance the efficacy of $\alpha$-lipoic acid as monotherapy toward preventing diabetic vascular and neural dysfunction. Treating diabetic rats with the combination of 0.25% $\alpha$-lipoic acid (in the diet) and fidarestat (3 mg/kg body weight) prevented the diabetes-induced slowing of motor nerve conduction velocity and endoneurial blood flow. This therapy also significantly improved acetylcholine-mediated vasodilation in epineurial arterioles of the sciatic nerve compared to nontreated diabetic rats. Treating diabetic rats with 0.25% $\alpha$-lipoic acid and fidarestat (3 mg/kg body weight) was equally or more effective in preventing vascular and neural dysfunction than was monotherapy of diabetic rats with higher doses of $\alpha$-lipoic acid or fidarestat. Treating diabetic rats with the combination of 0.25% $\alpha$-lipoic acid and fidarestat (3 mg/kg body weight) significantly improved several markers of oxidative stress and increased the serum levels of both $\alpha$-lipoic acid and dihydrolipoic acid. These studies suggest that combination therapy consisting of $\alpha$-lipoic acid and fidarestat may be more efficacious in preventing diabetes-induced vascular and neural dysfunction in peripheral tissue compared to monotherapy, which requires higher doses to be equally effective. The effect of this combination therapy may in part be due to the increased production and/or level of dihydrolipoic acid.**

**Keywords** $\alpha$-Lipoic Acid; Acetylcholine; Aldose Reductase; Diabetes; Diabetic Neuropathy; Endothelium; Fidarestat; Oxygen Radicals; Vasodilation

Received 4 August 2003; accepted 3 October 2003.

This work was supported by a National Institute of Diabetes and Digestive and Kidney Diseases grant DK-58005 from NIH, by a Merit Review grant from the Veterans Affairs Administration, and by the Sanwa Kagaku Kenkyusho Co., Ltd. (Japan). The contents of this manuscript are solely the responsibility of the authors and do not necessarily represent the official views of the NIH.

Address correspondence to Mark A. Yorek, 3 E 17 Veterans Affairs Medical Center, Iowa City, IA 52246, USA. E-mail: myorek@icva.gov

Diabetic neuropathy is a multifactorial problem likely due to the poor control of hyperglycemia. Two of the hyperglycemia-induced perturbations are increased oxidative stress and the activation of the polyol pathway [1, 2]. In animal models of diabetes, prevention of these defects by treatment with antioxidants or aldose reductase inhibitors has been shown to improve peripheral neuropathy [3–24].

Oxidative stress has been defined as a disturbance in the balance between the production of reactive oxygen species (oxygen free radicals, i.e., hydroxyl radical [$OH^{\cdot}$], superoxide anion [$O_2^{\cdot -}$], and hydrogen peroxide [$H_2O_2$]) and antioxidant defenses [25]. Oxidative stress and the damage that it causes have been implicated in a wide variety of natural and pathological processes, including aging, cancer, diabetes, atherosclerosis, neurological degeneration, schizophrenia, and autoimmune disorders such as arthritis [26]. Oxidative stress can be derived from a variety of sources and includes events such as the production of reactive oxygen species by NAD(P)H and xanthine oxidases, uncoupled nitric oxide synthase, mitochondrial oxidative phosphorylation, ionizing radiation exposure, and metabolism of exogenous compounds [26]. In addition to these sources of oxidative stress, a decrease in the activity of antioxidant enzymes, such as superoxide dismutase, glutathione peroxidase, and catalase, may contribute to oxidative stress in some disease states [27]. There is considerable evidence suggesting that oxidative stress occurs during the course of diabetes mellitus and contributes to the development of vascular and neural dysfunction [3–16]. Previously, we have shown that acetylcholine-induced vasodilation by arterioles that provide circulation to the region of the sciatic nerve is impaired early in diabetes and is accompanied by a reduction of endoneurial blood flow (EBF) and an increase in superoxide levels in these vessels [28, 29]. We have also demonstrated that $\alpha$-lipoic acid provides good protection against oxidative stress in diabetic rats for a 4- to 6-week duration, preventing both vascular and neural diabetes-induced impairments [30].

Studies from numerous laboratories have shown that treating diabetic rats with an aldose reductase inhibitor improves nerve function as well as EBF [17–24]. Aldose reductase inhibitor treatments have also been shown to prevent the depression of endothelium-dependent aortic relaxations induced by diabetes and abnormalities in contraction, and improve the synthesis of prostacyclin by the aorta of diabetic rats [31–33]. Recently, Keegan and colleagues have demonstrated that treating diabetic rats with the aldose reductase inhibitor WAY121509 completely prevented the diabetes-induced decrease in acetylcholine-induced relaxation in the corpus cavernosum vascular bed [34]. Previously, we have demonstrated that treating streptozotocin-induced diabetic rats with an aldose reductase inhibitor prevented metabolic derangements in the sciatic nerve, as well as the reduction of glutathione in the lens, and significantly improved endothelium-dependent vascular relaxation in epineurial arterioles of the sciatic nerve, motor nerve conduction velocity (MNCV), and EBF.

$\alpha$-Lipoic acid is a good metal chelator and is capable of scavenging hydroxyl radicals, hypochlorous acid, and singlet oxygen, but not superoxide or peroxyl radicals [35–38]. However, in its reduced form, as dihydrolipoic acid, it is a good scavenger of superoxide and prevents initiation of lipid peroxidation [35–38]. In vivo, $\alpha$-lipoic acid can be converted into dihydrolipoic acid [37, 38]. This reaction requires either NADH via a mitochondrial pathway or NADPH via cytosolic pathways [38, 39]. The latter cofactor is reduced in diabetes due to the increased flux of glucose through the aldose reductase pathway [40, 41]. In the present study, we sought to determine whether combining an aldose reductase inhibitor with $\alpha$-lipoic acid promotes the formation of dihydrolipoic acid, thereby enhancing the antioxidant potential of $\alpha$-lipoic acid.

### MATERIALS AND METHODS

Unless stated otherwise, all chemicals used in these studies were obtained from Sigma Chemical (St. Louis, MO). Fidarestat was a generous gift from Sanwa Kagaku Kenkyusho (Japan).

### Animals

Male Sprague-Dawley (Harlan Sprague-Dawley, Indianapolis, IN) rats, 8 to 9 weeks of age, were housed in a certified animal care facility and food (Harlan Teklad, no. 7001, Madison, WI) and water were provided ad libitum. All institutional and National Institutes of Health (NIH) guidelines for use of animals were followed. Diabetes was induced by intravenously injecting streptozotocin (55 mg/kg in 0.9% NaCl, adjusted to a pH 4.0 with 0.2 M sodium citrate). Control rats were injected with vehicle alone. The rats were anesthetized with halothane before injection. Diabetes was verified 48 hours later by evaluating blood glucose levels with the use of glucose oxidase reagent strips (Lifescan, Milpitas, CA). Rats having blood glucose levels of 300 mg/dL (16.7 mM) or greater were considered to be diabetic. At this time, the diabetic rats were randomly divided into 7 groups. Three of the groups received $\alpha$-lipoic acid (DL-6,8-thioctic acid) as a dietary supplement 0.1%, 0.25%, and 0.5% by weight. A 4th group received fidarestat, as a dietary supplement 3 mg/kg body weight (BW). The 5th and 6th group received a combination of fidarestat (3 mg/kg BW) and 0.1% or 0.25%, $\alpha$-lipoic acid by weight, respectively, as a dietary supplement. The 7th group represented the untreated diabetic group. In a separate experiment, another group of diabetic rats were treated with fidarestat (15 mg/kg BW). Based on diabetic rats daily consumption, the amount of $\alpha$-lipoic acid received on a daily basis by diabetic rats fed 0.25% $\alpha$-lipoic acid in the diet was 175 mg/kg/rat. In studies conducted by Cameron and colleagues, rats were given racemate $\alpha$-lipoic acid in the diet and the calculated daily dose that the rats received was about 300 mg/kg/day [10]. The $\alpha$-lipoic acid and fidarestat were added to a meal form of the diet that was formed into pellets for feeding purposes. Analysis of the diet containing $\alpha$-lipoic acid revealed undetectable levels of dihydrolipoic acid. Control and untreated diabetic rats were fed nonsupplemented pelleted rat chow. The chow was made in the laboratory, dried in a vacuum oven, and stored refrigerated until use. All studies were conducted approximately 4 to 5 weeks after the verification of diabetes.

On the day of the experiment, blood glucose level was determined and the rats were anesthetized with Nembutal intraperitoneally (50 mg/kg, intraperitoneal [IP]; Abbott Laboratories, North Chicago, IL). Following the determination of MNCV and EBF, the abdominal aorta was isolated and occluded 1 to 2 cm above the branch of the common iliac artery. The rat was then sacrificed by exsanguination, and body temperature lowered with topical ice. Samples of the left sciatic nerve were then taken for determination of $Na^+$, $K^+$-ATPase activity and sorbitol, fructose, and *myo*-inositol content. For markers of oxidative stress, we collected the lens for determination of glutathione levels, the aorta was collected for the determination of superoxide, and serum samples were collected for determination of thiobarbituric acid–reactive substances (TBARSs).

### Motor Nerve Conduction Velocity

MNCV was determined as previously described using a noninvasive procedure in the sciatic-posterior tibial conducting system in a temperature-controlled environment [28–30]. The left sciatic nerve was stimulated first at the sciatic notch and then at the Achilles tendon. Stimulation consisted of single 0.2-ms supramaximal (8 V) pulses through a bipolar electrode (Grass S44 Stimulator, Grass Medical Instruments, Quincy, MA). The evoked potentials were recorded from the interosseous muscle with a unipolar platinum electrode and displayed on a digital storage oscilloscope (model 54600A; Hewlett Packard, Rolling Meadows, IL). MNCV was calculated by subtracting the distal from the proximal latency measured in milliseconds from the stimulus artifact of the take-off of the evoked potential and the difference was divided into the distance between the 2 stimulating electrodes measured in millimeters using a Vernier caliper. The MNCV was reported in meters per second.

### Endoneurial Blood Flow

Immediately after determination of MNCV, sciatic nerve endoneurial nutritive blood flow was determined as previously described [28–30]. The trachea was intubated for mechanical ventilation and a carotid cannula inserted to monitor mean arterial blood pressure. Core temperature was monitored using a rectal probe and temperature regulated between 36°C and 37°C using a heating pad and radiant heat. The right sciatic nerve was carefully exposed by a small surgical incision and the surrounding skin sutured to a plastic ring. The isolated area was filled with mineral oil at 37°C to a depth of 1 cm to minimize diffusion of hydrogen gas from the nerve. The rats were then mechanically ventilated. A glass insulated platinum microelectrode (tip = 2 $\mu$m) was inserted into the sciatic nerve, proximal to the trifurcation, and polarized at 0.25 V with respect to a reference electrode inserted subcutaneously into the flank of the rat. Once the recording had stabilized, the inspired air was modified to contain 10% hydrogen gas and this gas flow continued until the hydrogen current recorded by the electrode had stabilized, indicating equilibrium of the inspired air with arterial blood. The hydrogen gas supply was then discontinued and the hydrogen clearance curve recorded until a baseline was achieved. The hydrogen clearance data were fitted to a mono- or biexponential curve using commercial software (Prism; GraphPad, San Diego, CA) and nutritive blood flow, (mL/min/100 g), calculated using the equation described by Young [42], and vascular conductance (mL/min/100 g/mm Hg), determined by dividing nutritive blood flow by the average mean arterial blood pressure. Two recordings were made for each rat at different locations along the nerve and the final blood flow value averaged.

### Vascular Reactivity

Videomicroscopy was used to investigate in vitro vasodilatory responsiveness of arterioles vascularizing the region of the sciatic nerve (branches of the superior gluteal and internal pudendal arteries) as previously described [28–30]. The vessels used for these studies were generally oriented longitudinally in relation to the sciatic nerve; however, on occasion radially oriented vessels were also used. No differences were observed in acetylcholine-induced vasodilation based on the orientation of the vessel to the sciatic nerve. The arterioles used in this study should be regarded as epineurial rather than perineurial vessels. To isolate these vessels, the common iliac was exposed and the branch points of the internal pudendal and superior gluteal arteries identified. The vessels were then clamped, and tissue containing these vessels and its branches dissected en bloc. The block of tissue was immediately submerged in a cooled (4°C), oxygenated (20% $O_2$, 5% $CO_2$, and 75% $N_2$) Krebs Henseleit physiological saline solution (PSS) of the following composition (in mM): NaCl 118, KCl 4.7, $CaCl_2$ 2.5, $KH_2PO_4$ 1.2, $MgSO_4$ 1.2, $NaHCO_3$ 20, $Na_2EDTA$ 0.026, and glucose 5.5. Branches of the superior gluteal and internal pudendal arteries (50 to 150 $\mu$m internal diameter and 2 mm in length) were carefully dissected and trimmed of fat and connective tissue. Both ends of the isolated vessel segment were cannulated with glass micropipettes filled with PSS (4°C), and secured with 10-0 nylon Ethilon monofilament sutures (Ethicon, Cornelia, GA). The pipettes were attached to a single pressure reservoir (initially set at 0 mm Hg) under condition of no flow. The organ chamber containing the cannulated vessels was then transferred to the stage of an inverted microscope (CK2; Olympus, Lake Success, NY). Attached to the microscope were a CCTV camera (WV-BL200; Panasonic, Secaucus, NJ), video monitor (Panasonic), and a video caliper (VIA-100 K; Boeckeler Instruments, Tucson, AZ). The organ chamber was connected to a rotary pump (Masterflex; Cole Parmer Instrument, Vernon Hills, IL), which continuously circulated 37°C oxygenated PSS at 30 mL/min. The pressure within the vessel was then slowly increased to 40 mm Hg. At this pressure, we found that KCl gave the maximal constrictor response. Therefore, all the studies were conducted at 40 mm Hg. Internal vessel diameter (resolution of 2 $\mu$m) was measured by manually adjusting the video micrometer. After 30 minutes' equilibration, KCl was added to the bath to test vessel viability. Vessels, which failed to constrict more than 30%, were discarded. After washing with PSS, vessels were incubated for 30 minutes in PSS and then constricted with U46619 ($10^{-8}$ to $10^{-7}$ M) (Cayman Chemical,

Ann Arbor, MI) to 30% to 50% of passive diameter. There was no significant difference in the amount of U46619 required to induce constriction in control and diabetic vessels. Cumulative concentration-response relationships were evaluated for acetylcholine ($10^{-8}$ to $10^{-4}$ M) using vessels from control and diabetic rats. At the end of each acetylcholine dose-response determination, sodium nitroprusside ($10^{-4}$ M) was added to determine maximal vasodilation.

### Detection of Superoxide

Superoxide levels were measured using the aorta by lucigenin-enhanced chemiluminescence as described previously [30]. Vessel segments were incubated in 0.5 mL phosphate-buffered saline containing lucigenin (5 $\mu$mol/L) and afterwards, relative light units (RLUs) measured using a Zylux FB12 luminometer. For these studies, chemiluminescence was measured for 5 minutes. Background activities were determined and subtracted and RLUs were normalized to surface area.

### Sciatic Nerve Sorbitol, Fructose, and *myo*-Inositol Content

As a marker of fidarestat treatment efficacy, the levels of sciatic nerve sorbitol, fructose, and *myo*-inositol were determined. Briefly, the tissue samples were boiled for 10 minutes in water containing $\alpha$-D-methylmannopyranoside as an internal standard and deproteinized with 0.5 mL each of 0.19 M $Ba(OH)_2$ and 0.19 M $ZnSO_4$. Following centrifugation, the supernatant was collected, frozen, and lyophilized. Afterwards, the samples were derivatized and intracellular content of sorbitol and *myo*-inositol determined by gas-liquid chromatography as previously described [28–30].

### Additional Biological Parameters

Lens glutathione (GSH) and serum TBARS levels were determined as additional markers of oxidative stress. Lens glutathione levels were determined according to Lou and colleagues [43]. Lens were weighed and homogenized in 1 mL of cold 10% trichloroacetic acid and centrifuged for 15 minutes at 1000 $\times$ $g$. The supernatant (100 $\mu$L) was mixed with 0.89 mL of 1.0 M Tris, pH 8.2, and 0.02 M EDTA. Afterwards, 10 $\mu$L of dithionitrobenzene was added and change in absorbance measured at 412 nm. A glutathione standard curve (100 to 500 ng) was performed for each assay. The data were recorded as $\mu$g/mg wet weight. TBARS level in serum was determined by the method of Mihara and colleagues [44] as modified by Siman and Eriksson [45]. Briefly, 200 $\mu$L of serum was boiled in 0.75 mL of phosphoric acid (0.19 M), 0.25 mL thiobarbituric acid (0.42 mM), and 0.3 mL water for 60 minutes. Afterwards, the samples were precipitated with methanol/NaOH and centrifuged for 5 minutes. The supernatant was measured fluorometrically at excitation wavelength of 532 nm and emission wavelength of 553 nm. Standards were prepared by the acid hydrolysis of 1,1,3,3-tetraethoxypropane. The data were reported as $\mu$g/mL serum. Serum $\alpha$-lipoic, acid and dihydrolipoic acid levels were determined as described by Teichert and Preiss [46]. Serum, 0.5 mL, was acidified with 3 mL 0.05 M HCl. The acidified serum was then applied to a phenyl solid phase extraction column, which was washed with methanol and preequilibrated with 0.05 M HCl. The column was then rinsed with 4 mL 0.05 M HCl and eluted with 1:1 methanol:acetonitrile. The sample was dried under nitrogen, resuspended in 100 $\mu$L acetonitrile and injected onto the high-performance liquid chromatography (HPLC) column. Chromatography was carried out using a Neucleosil C-18 5-$\mu$ 120 A 250 $\times$ 4-mm reversed phase column. The column was eluted isocratically with 33% acetonitrile:67% 0.05 M potassium phosphate pH 2.9, at a flow rate of 1 mL/min. The eluent was monitored amperometrically using a glassy carbon electrode at a potential of 1.10 V. Concentrations were determined by comparison to a standard curve. Dihydrolipoic acid is easily and quickly oxidized to $\alpha$-lipoic acid, so care must be taken to eliminate oxygen whenever possible. All samples were treated identically and HPLC solvents were sparged with, and stored under, helium.

### Data Analysis

The results are presented as mean $\pm$ SE. Comparisons between the groups for MNCV; EBF; sciatic nerve $Na^+$,$K^+$-ATPase activity; sciatic nerve sorbitol, fructose, and *myo*-inositol content; and serum TBARS and lens glutathione levels were conducted using a 1-way analysis of variance (ANOVA) and Newman-Keuls test for multiple comparisons (Prism; GraphPad). Dose-response curves for acetylcholine-induced relaxation were compared using a 2-way repeated-measure ANOVA with autoregressive covariance structure using proc mixed program of SAS [28–30]. Whenever significant interactions were noted, specific treatment dose effects were analyzed using a Bonferroni adjustment. A $P$ value of less than .05 was considered significant.

### RESULTS

### Animal Statistics

Data in Table 1 provide information on the final body weight and blood glucose level for the rats used in these studies. Blood glucose level was significantly increased in all diabetic rats and the diabetic rats failed to gain weight compared to the control rats. There was no significant difference in blood glucose level and final body weight between the untreated diabetic rats and treated diabetic rats.

**TABLE 1**
Physiological parameters for control and diabetic rats

| Group | n | Final weight (g) | Blood glucose (mmol/L) |
|---|---|---|---|
| Control | 9 | 397 ± 18 | 5.1 ± 0.4 |
| Diabetic | 9 | 214 ± 17[a] | 25.6 ± 0.5[a] |
| D + 0.1% LA | 8 | 239 ± 22[a] | 21.3 ± 1.5[a] |
| D + 0.25% LA | 8 | 210 ± 16[a] | 22.6 ± 1.3[a] |
| D + 0.5% LA | 6 | 190 ± 20[a] | 21.0 ± 0.7[a] |
| D + 0.1% LA + Fid | 9 | 255 ± 17[a] | 20.4 ± 1.1[a] |
| D + 0.25% LA + Fid | 9 | 241 ± 15[a] | 22.2 ± 1.4[a] |
| D + Fid (3 mg/kg BW) | 10 | 262 ± 17[a] | 24.9 ± 0.7[a] |
| D + Fid (15 mg/kg BW) | 4 | 300 ± 12[a] | 21.6 ± 1.3[a] |

*Note.* Streptozotocin-induced diabetic rats (D) were treated with 0.1% to 0.5% α-lipoic acid (LA) in the diet and/or fidarestat (Fid), 3 mg/kg BW. A separate set of diabetic rats was treated with 15 mg/kg BW fidarestat. After 4 to 5 weeks of diabetes and with or without treatment, the studies were performed. Data are presented as mean ± SEM for each group.
[a] $P < .05$ compared to control.

### Parameters of Oxidative Stress

Serum TBARSs, lens glutathione, and superoxide content of the aorta were measured as markers of oxidative stress. Data in Table 2 demonstrate that diabetes causes a significant increase in serum TBARS and aorta superoxide levels and a decrease in lens glutathione level. Treating diabetic rats with α-lipoic acid (0.1% to 0.5%) significantly reduced superoxide in the aorta but had no effect on lens glutathione level. Treating rats with α-lipoic acid tended to reduce serum TBARS level but a significant decrease from diabetic rats was only observed with 0.25% α-lipoic acid. Treating diabetic rats with fidarestat alone at 3 mg/kg BW or 15 mg/kg BW significantly reduced aorta superoxide levels but not to the same extent as observed with treatment of α-lipoic acid. The low dose of fidarestat had no effect on serum TBARS or lens glutathione levels. In contrast, treating rats with 15 mg/kg BW restored lens glutathione level to normal values and partially corrected the diabetes-induced increase in serum TBARS level. Treatment of diabetic rats with the combination of 0.25% α-lipoic acid and fidarestat, 3 mg/kg BW, significantly decreased aorta superoxide and serum TBARS level and increased lens glutathione level. However, only the combination of 0.25% α-lipoic acid and 3 mg/kg BW fidarestat was more effective than monotherapy in restoring lens glutathione level. Data in Table 2 also present the changes in serum α-lipoic acid and dihydrolipoic acid levels. Treating diabetic rats with α-lipoic acid (0.1% to 0.5%) did not significantly increase serum α-lipoic acid or dihydrolipoic acid levels compared to control or untreated diabetic rats. Treatment of diabetic rats with the combination of 0.25% α-lipoic acid and fidarestat, 3 mg/kg BW, caused a significant increase in both serum α-lipoic acid and dihydrolipoic acid levels compared to control, untreated diabetic rats, or diabetic rats treated with 0.25% α-lipoic acid or 3 mg/kg BW fidarestat alone.

**TABLE 2**
Determination of serum TBARS, lens GSH, aorta superoxide, and serum α-lipoic acid (LA) and dihydrolipoic acid (DHLA) in control and diabetic rats

| Group | TBARS (μg/mL) | GSH (μg/mg wet wt) | Superoxide (RLU/min/mm$^2$) | LA/DHLA (pmol/mL) |
|---|---|---|---|---|
| Control | 0.39 ± 0.03 | 3.0 ± 0.3 | 1.99 ± 0.26 | 15.5 ± 2.1/1.9 ± 0.2 |
| Diabetic | 0.55 ± 0.03[a] | 1.1 ± 0.1[a] | 3.38 ± 0.26[a] | 15.2 ± 2.2/1.0 ± 0.2 |
| D + 0.1% LA | 0.46 ± 0.05 | 1.1 ± 0.1[a] | 1.94 ± 0.23[b] | 15.4 ± 4.6/1.1 ± 0.2 |
| D + 0.25% LA | 0.40 ± 0.02[b] | 1.2 ± 0.1[a] | 1.68 ± 0.15[b] | 12.7 ± 1.0/3.4 ± 0.3 |
| D + 0.5% LA | 0.45 ± 0.03 | 1.1 ± 0.1[a] | 1.66 ± 0.12[b] | 15.8 ± 0.8/3.3 ± 0.3 |
| D + 0.1% LA + Fid | 0.39 ± 0.03[b,d] | 2.2 ± 0.3[b,c] | 2.20 ± 0.33[b] | 27.8 ± 4.3[d]/4.4 ± 0.5[d] |
| D + 0.25% LA + Fid | 0.38 ± 0.02[b,d] | 2.7 ± 0.4[b,c,d] | 1.86 ± 0.19[b] | 35.7 ± 2.6[a,b,c,d]/17.9 ± 2.1[a,b,c,d] |
| D + Fid (3 mg/kg BW) | 0.59 ± 0.05[a] | 1.5 ± 0.2[a] | 2.28 ± 0.28[b] | 9.6 ± 1.2/0.9 ± 0.3 |
| D + Fid (15 mg/kg BW) | 0.47 ± 0.09 | 3.7 ± 0.5[b] | 2.30 ± 0.26[b] | ND |

*Note.* Treatment conditions and the number of experimental observations were the same as described in Table 1. Data are presented as the mean ± SEM for each group. Data for aorta superoxide are presented as relative light units (RLU).
[a] $P < .05$ compared to control.
[b] $P < .00$ compared to untreated diabetic.
[c] $P < .05$ compared to D + LA.
[d] $P < .05$ compared to D + Fid (3 mg/kg).
ND = not determined.

**TABLE 3**
Determination of motor nerve conduction velocity (MNCV) and endoneurial blood flow (EBF) in control and diabetic rats

| Group | MNCV (m/s) | EBF nutritive/conductance mL/min/100 g | mL/min/100 g/mm Hg |
|---|---|---|---|
| Control | 56.7 ± 1.8 | 25.8 ± 5.7 | 0.195 ± 0.031 |
| Diabetic | 38.2 ± 1.1[a] | 7.6 ± 1.1[a] | 0.065 ± 0.011[a] |
| D + 0.1% LA | 47.2 ± 2.2[a,b] | 19.0 ± 4.3 | 0.144 ± 0.023 |
| D + 0.25% LA | 47.3 ± 2.3[a,b] | 24.4 ± 4.4[b] | 0.198 ± 0.026[b] |
| D + 0.5% LA | 49.5 ± 1.5[b] | 23.0 ± 2.4[b] | 0.191 ± 0.044[b] |
| D + 0.1% LA + Fid | 48.2 ± 2.2[a,b] | 16.9 ± 2.9 | 0.125 ± 0.017 |
| D + 0.25% LA + Fid | 51.6 ± 1.5[b] | 26.4 ± 4.2[b] | 0.202 ± 0.013[b] |
| D + Fid (3 mg/kg BW) | 48.7 ± 1.9[a,b] | 16.1 ± 5.1 | 0.127 ± 0.031 |
| D + Fid (15 mg/kg BW) | 53.7 ± 1.4[b] | 22.8 ± 4.1[b] | 0.175 ± 0.013[b] |

*Note*. Treatment conditions and the number of experimental observations were the same as described in Table 1. Data are presented as the mean ± SEM.
[a] $P < .05$ compared to control.
[b] $P < .05$ compared to untreated diabetic.

### Neural Parameters

Diabetes caused a significant impairment in sciatic nerve EBF and MNCV (Table 3). Treating diabetic rats with $\alpha$-lipoic acid (0.25% and 0.5%) or 15 mg/kg BW fidarestat alone significantly improved sciatic nerve EBF compared to untreated diabetic rats. Treating diabetic rats with 0.1% $\alpha$-lipoic acid or 3 mg/kg BW fidarestat did not significantly improve sciatic nerve EBF compared to untreated diabetic rats and the combination therapy of $\alpha$-lipoic acid and 3 mg/kg BW fidarestat was not significantly more effective in improving sciatic nerve EBF than monotherapy with $\alpha$-lipoic acid. MNCV was significantly improved compared to untreated diabetic rats by either the monotherapy of $\alpha$-lipoic acid (0.1% to 0.5%) or fidarestat (3 or 15 mg/kg BW) or the combination therapy. The greatest improvement was observed with the combination therapy of 0.25% $\alpha$-lipoic acid and 3 mg/kg BW fidarestat or 15 mg/kg BW fidarestat alone.

### Vascular Reactivity of Epineurial Arterioles of the Sciatic Nerve

Figures 1 to 4 provide data on the influence of these treatment strategies on acetylcholine-mediated vasodilation by epineurial arterioles of the sciatic nerve. We previously reported that treating streptozotocin-induced diabetic rats with $\alpha$-lipoic acid using a prevention protocol prevented in a concentration-dependent manner the diabetes-induced impairment in acetylcholine-mediated vasodilation [30]. These results are confirmed from studies presented in Figure 1. Treating diabetic rats with $\alpha$-lipoic acid, 0.5%, was most effective in preventing the diabetes-induced impairment in vasodilation mediated by acetylcholine compared to diabetic rats treated with 0.1% and 0.25% $\alpha$-lipoic acid. We had also previously reported that treating diabetic rats with an aldose reductase inhibitor significantly improved acetylcholine-mediated vasodilation in epineurial arterioles of the sciatic nerve [47]. In these studies, we examined the effect of the combination therapy of fidarestat, an aldose reductase inhibitor, and $\alpha$-lipoic acid on acetylcholine-mediated vasodilation in epineurial arterioles. In order to determine the effect of this combination of treatments, the dose of fidarestat (3 mg/kg BW) used in these studies was one that showed minimal effect when used alone in improving vascular function, as evident by the small improvement in EBF (Table 3) and acetylcholine-mediated vasodilation (Figure 2). In contrast, when a higher dose of fidarestat was used (15 mg/kg BW) in treating diabetic rats, the improvement in acetylcholine-mediated vasodilation was significant compared to untreated diabetic rats and diabetic rats treated with 3 mg/kg BW fidarestat, and similar to the benefits reported by us for sorbinil treatment of diabetic rats [47]. Figures 3 and 4 show the data for treating diabetic rats with 0.1% $\alpha$-lipoic acid plus fidarestat (3 mg/kg BW) and 0.25% $\alpha$-lipoic acid plus fidarestat (3 mg/kg BW), respectively. Data in Figure 3 demonstrate that treating diabetic rats with 0.1% $\alpha$-lipoic acid alone has minimal benefit on preventing the diabetes-induced impairment in acetylcholine-mediated vasodilation in epineurial arterioles. In contrast, combining 0.1% $\alpha$-lipoic acid with fidarestat (3 mg/kg BW) significantly improves acetylcholine-mediated vasodilation compared to diabetic rats. However, acetylcholine-mediated vasodilation in diabetic rats treated with 0.1% $\alpha$-lipoic acid with or without fidarestat (3 mg/kg BW) remained significantly impaired compared to control rats.

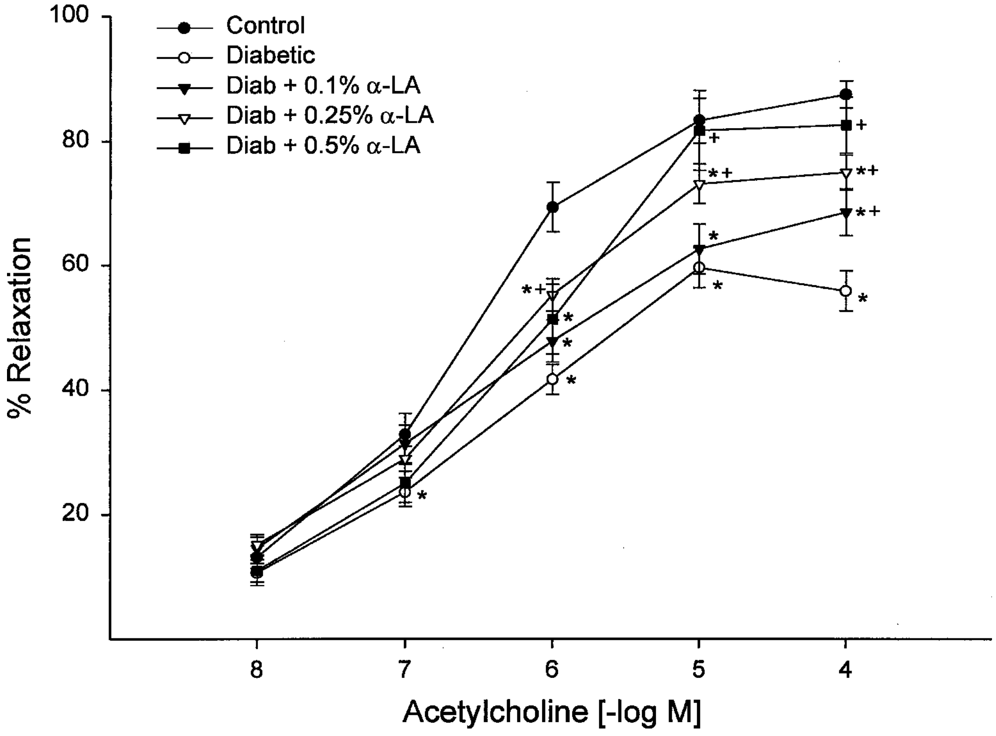

**FIGURE 1**

Determination of the effect of treatment of diabetic rats (Diab) with 0.1% to 0.5% $\alpha$-lipoic acid ($\alpha$-LA) on acetylcholine-mediated vascular relaxation in epineurial arterioles of the sciatic nerve. Pressurized arterioles were preconstricted with U46619 (30% to 50%), and incremental doses of acetylcholine were added to the bathing solution while the steady-state vessel diameter was recorded. The number of experimental animals used in these studies was the same as that noted in Table 1. *The response to acetylcholine was significantly different compared to control; +the response to acetylcholine was significantly different compared to the untreated diabetic rats.

Treating diabetic rats with 0.25% $\alpha$-lipoic acid significantly improved acetylcholine-mediated vasodilation in epineurial arterioles and was more efficacious than treatment with 0.1% $\alpha$-lipoic acid (Figure 4). Nonetheless, acetylcholine-mediated vasodilation in epineurial arterioles from diabetic rats treated with 0.25% $\alpha$-lipoic acid remained significantly impaired compared to control rats. Treating diabetic rats with 0.25% $\alpha$-lipoic acid and fidarestat (3 mg/kg BW) normalized acetylcholine-mediated vasodilation in epineurial arterioles and was significantly improved compared to diabetic rats treated with 0.25% $\alpha$-lipoic acid alone. Furthermore, treating diabetic rats with 0.25% $\alpha$-lipoic acid and fidarestat (3 mg/kg BW) was more efficacious than treatment of diabetic rats with 0.5% $\alpha$-lipoic acid or 15 mg/kg BW fidarestat in the diet alone on acetylcholine-mediated vasodilation.

### DISCUSSION

Previously, we had reported that treating streptozotocin-induced diabetic rats with 0.5% $\alpha$-lipoic acid provides maximum protection against diabetes-induced oxidative stress and the development of vascular and neural dysfunction in peripheral tissues [30]. We have also reported that sorbinil, an aldose reductase inhibitor, partially prevents the development of diabetes-induced vascular and neural defects but was not as efficacious as antioxidant therapies [30, 47]. In the present study, we found that combining these therapies maximizes the benefits in preventing diabetes-induced slowing of MNCV, EBF, oxidative stress, and vascular dysfunction. The combination of 0.25% $\alpha$-lipoic acid (in the diet) and fidarestat (3 mg/kg BW) completely prevented the diabetes-induced impairment of acetylcholine-mediated vascular relaxation in epineurial arterioles of the sciatic nerve. This combination was more effective in preventing diabetes-induced vascular dysfunction than monotherapy of either compound when given at higher doses.

One possible explanation for these results is that treatment of diabetic rats with fidarestat in combination with $\alpha$-lipoic acid favors the formation of dihydrolipoic acid. $\alpha$-Lipoic acid is a good metal chelator and is capable of scavenging hydroxyl radicals, hypochlorous acid, and singlet oxygen, but not superoxide

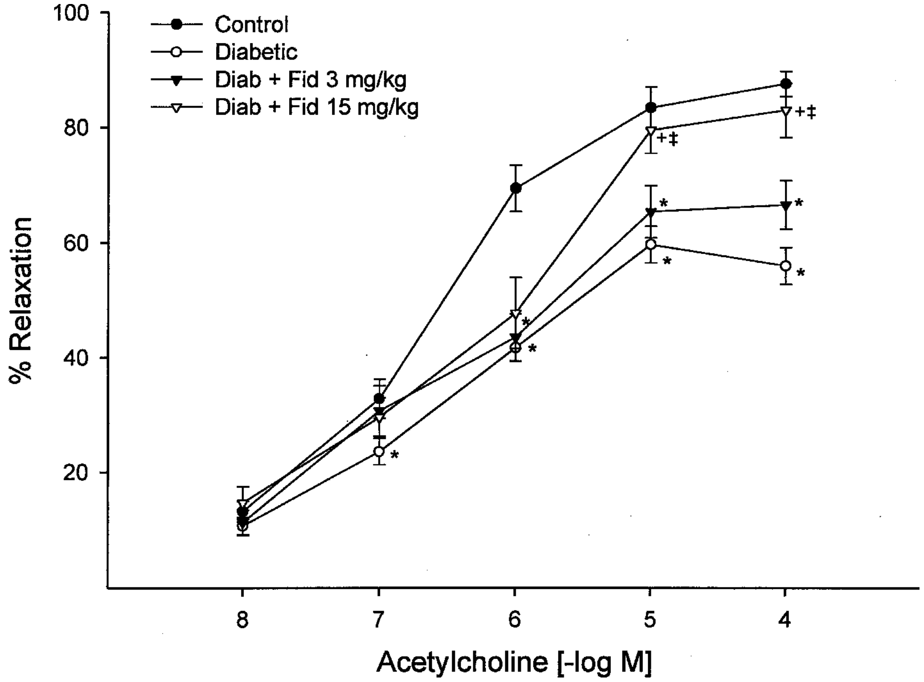

**FIGURE 2**
Determination of the effect of treatment of diabetic rats (Diab) with fidarestat (Fid) (3 mg/kg BW) or fidarestat (15 mg/kg BW) on acetylcholine-mediated vascular relaxation in epineurial arterioles of the sciatic nerve. Pressurized arterioles were preconstricted with U46619 (30% to 50%), and incremental doses of acetylcholine were added to the bathing solution while the steady-state vessel diameter was recorded. The number of experimental animals used in these studies was the same as that noted in Table 1. *The response to acetylcholine was significantly different compared to control; +the response to acetylcholine was significantly different compared to the untreated diabetic rats; ‡the response to acetylcholine was significantly different compared to the 3-mg/kg BW fidarestat-treated diabetic rats.

or peroxyl radicals [35–38]. However, in its reduced form, as dihydrolipoic acid, it is a good scavenger of superoxide and prevents initiation of lipid peroxidation [35–38]. In vivo, the conversion of $\alpha$-lipoic acid to dihydrolipoic acid requires either NADH or NADPH [37, 38]. In the mitochondria, preferentially $R(+)$-$\alpha$-lipoic acid is converted to dihydrolipoic acid by the action of dihydrolipoamide dehydrogenase which requires NADH [38, 39]. Both stereoisoforms of $\alpha$-lipoic acid can be reduced in the cytosol by glutathione reductase or thioredoxin reductase, and both require NADPH [38, 39, 48]. In neutrophils, as well as rat heart, kidney, and brain, NADH-dependent reduction of $\alpha$-lipoic acid is prominent, whereas with rat liver, NADH- and NADPH-dependent pathways were about equally active [38, 39]. In erythrocytes and endothelial cells, NADPH is the primary reducing cofactor for $\alpha$-lipoic acid [38, 49]. In diabetes, NADPH levels are reduced due to the increased flux of glucose through the aldose reductase pathway [40, 41]. Therefore, blocking the aldose reductase pathway with aldose reductase inhibitors such as fidarestat likely restores NADPH levels, permitting the formation of dihydrolipoic acid. This explanation is supported by our studies demonstrating that serum dihydrolipoic acid levels are increased in diabetic rats treated with 0.25% $\alpha$-lipoic acid and fidarestat (3 mg/kg BW). It is unlikely that treating diabetic rats with fidarestat would promote the formation of dihydrolipoic acid by the mitochondrial pathway because dihydrolipoamide dehydrogenase requires NADH [38, 39, 50]. Interestingly, the level of $\alpha$-lipoic acid in serum was also increased in diabetic rats treated with 0.25% $\alpha$-lipoic acid and fidarestat (3 mg/kg BW) compared to diabetic rats treated with 0.25% $\alpha$-lipoic acid alone. The reason for this is unknown but could be due to increased stability of the metabolism of $\alpha$-lipoic acid in the presence of aldose reductase inhibition. We do not know the cellular source of the increased serum levels of dihydrolipoic acid observed in our studies or the content of $\alpha$-lipoic acid or dihydrolipoic acid existing in the various tissues. The latter could have an impact on the serum levels of either $\alpha$-lipoic acid or dihydrolipoic acid if one or the other is preferentially taken up by tissues.

For these studies, we used a low concentration of fidarestat in order to detect whether the combination of $\alpha$-lipoic acid and

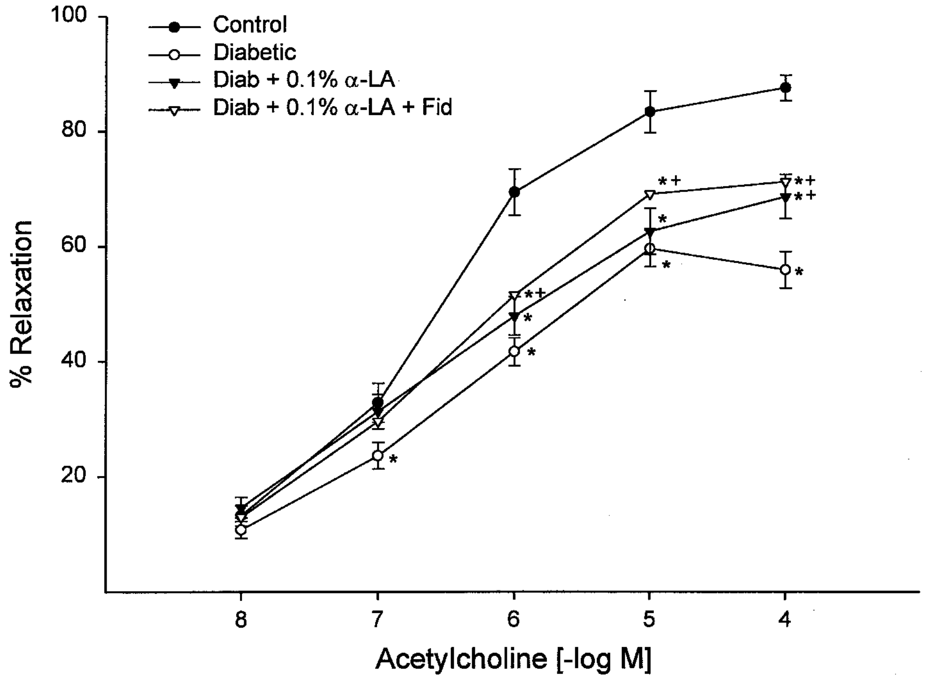

**FIGURE 3**

Determination of the effect of treatment of diabetic rats (Diab) with 0.1% $\alpha$-lipoic acid ($\alpha$-LA) with or without fidarestat (Fid) (3 mg/kg BW) on acetylcholine-mediated vascular relaxation in epineurial arterioles of the sciatic nerve. Pressurized arterioles were preconstricted with U46619 (30% to 50%), and incremental doses of acetylcholine were added to the bathing solution while the steady-state vessel diameter was recorded. The number of experimental animals used in these studies was the same as that noted in Table 1. *The response to acetylcholine was significantly different compared to control; +the response to acetylcholine was significantly different compared to the untreated diabetic rats.

fidarestat had additive effects on diabetes-induced vascular and neural dysfunction. At 3 mg/kg BW of fidarestat, fructose and sorbitol formation from the aldose reductase pathway was reduced by 75% and 70%, respectively, and in the sciatic nerve of diabetic rats compared to the sciatic nerve from untreated diabetic rats (data not shown). However, compared to control animals, the levels of fructose and sorbitol in the sciatic nerve from fidarestat-treated (3 mg/kg BW) diabetic rats remained significantly increased. In contrast, treatment of diabetic rats with 15 mg/kg BW fidarestat normalized fructose and sorbitol levels in the sciatic nerve (data not shown). Therefore, the dose of fidarestat used in the $\alpha$-lipoic acid and fidarestat combination studies was suboptimal based on efficacy as determined by fructose and sorbitol formation in the sciatic nerve of diabetic rats. At 3 mg/kg BW, fidarestat was also suboptimal in restoring lens glutathione levels and was only partially effective in preventing superoxide formation in the aorta. Treating diabetic rats with 15 mg/kg BW fidarestat normalized glutathione levels in the lens. These studies demonstrate that in addition to protecting glutathione production, treatment of diabetic rats with fidarestat in combination with $\alpha$-lipoic acid may promote the formation of dihydrolipoic acid. This result may explain the antioxidant properties of aldose reductase inhibitors [51].

In these studies, the effect of $\alpha$-lipoic acid and fidarestat treatment to protect EBF, MNCV, and lens glutathione levels as well as reduce the generation of superoxide appeared to be independent of any additive influence of the two treatments. Independently, treatment of diabetic rats with $\alpha$-lipoic acid in a concentration-dependent manner improved EBF and acetylcholine-mediated vasodilation in epineurial arterioles. There appeared to be no concentration-dependent effect by $\alpha$-lipoic acid in improving MNCV. $\alpha$-Lipoic acid at 0.1% in the diet was nearly as effective as 0.5% $\alpha$-lipoic acid in improving MNCV. This suggests that the diabetes-induced defect in MNCV may be due to several derangements and not solely mediated by a reduction in EBF and impaired vascular reactivity. This is not a new concept, and lends support to the idea that combination therapy may be the most effective means of preventing diabetic neuropathy. Treating diabetic rats with 0.25% $\alpha$-lipoic acid reduced the formation of superoxide in the aorta and serum TBARSs to the same degree as the combination treatment of 0.25% $\alpha$-lipoic acid and 3 mg/kg

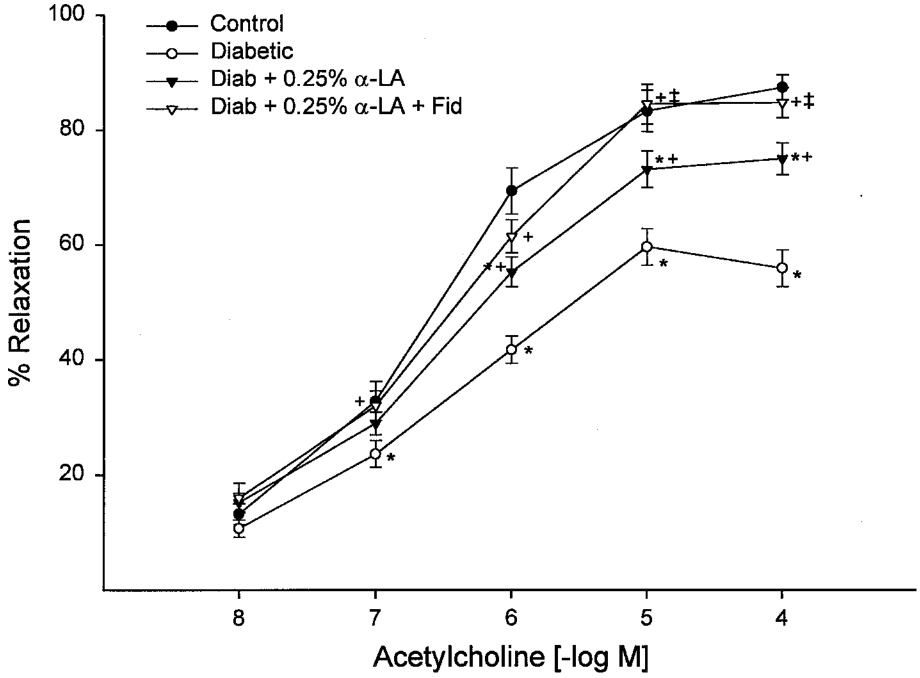

**FIGURE 4**

Determination of the effect of treatment of diabetic rats (Diab) with 0.25% $\alpha$-lipoic acid ($\alpha$-LA) with or without fidarestat (Fid) (3 mg/kg BW) on acetylcholine-mediated vascular relaxation in epineurial arterioles of the sciatic nerve. Pressurized arterioles were preconstricted with U46619 (30–50%), and incremental doses of acetylcholine were added to the bathing solution while the steady-state vessel diameter was recorded. The number of experimental animals used in these studies was the same as that noted in Table 1. *The response to acetylcholine was significantly different compared to control; +the response to acetylcholine was significantly different compared to the untreated diabetic rats; ‡the response to acetylcholine was significantly different compared to the 0.25% $\alpha$-lipoic acid or fidarestat (3 mg/kg BW)-treated diabetic rats.

fidarestat. In our studies, treating diabetic rats with $\alpha$-lipoic acid alone had no effect on improving glutathione levels in the lens [30]. These results differ from studies conducted by Stevens and colleagues [52]. In those studies, treating diabetic rats with 100 mg/kg/day IP $\alpha$-lipoic acid corrected glutathione levels in the sciatic nerve. In our studies, rats were treated with 0.25% $\alpha$-lipoic acid in the diet. Based on the average daily consumption, our rats received about 175 mg/kg/day $\alpha$-lipoic acid. The difference in these results could be due to glutathione measurements being conducted in different tissues or the different delivery methods for $\alpha$-lipoic acid treatment. We did find that treating diabetic rats with 15 mg/kg fidarestat improved lens glutathione levels. This is similar to the effect of sorbinil treatment of diabetic rats on lens glutathione levels we reported previously [47]. In these studies, there was a synergistic effect on improving lens glutathione levels when treating diabetic rats with the combination of 0.25% $\alpha$-lipoic acid and 3 mg/kg BW fidarestat. Treatment of diabetic rats with fidarestat (3 mg/kg BW) alone independently improved EBF and MNCV, by 50% and 60%, respectively, and reduced superoxide formation in the aorta. Furthermore, treating diabetic rats with 3 or 15 mg/kg BW of fidarestat had a concentration-dependent effect on improving EBF, MNCV, and acetylcholine-mediated vasodilation in epineurial arterioles. Taken together, our results imply that some markers of oxidative stress and neural function are significantly improved by monotherapy using $\alpha$-lipoic acid; however, the greatest beneficial effects were observed on all markers of oxidative stress and vascular function when the combination treatment consisting of 0.25% $\alpha$-lipoic acid and 3 mg/kg BW fidarestat was used.

In these studies, the combination of 0.25% $\alpha$-lipoic acid and fidarestat (3 mg/kg BW) provided the most efficient improvement in EBF, MNCV, and acetylcholine-mediated vasodilation in epineurial arterioles. There did not appear to be any additive-like interaction of these compounds in correcting EBF or MNCV, although the improvement of acetylcholine-mediated vascular relaxation by epineurial arterioles from diabetic rats did demonstrate additive-like responsiveness to the combination therapy. Treating diabetic rats with 0.25% $\alpha$-lipoic acid and fidarestat (3 mg/kg BW) significantly improved vasodilation in response to acetylcholine compared to monotherapy. In addition, the effectiveness of the combination therapy in improving vascular reactivity was greater than the improvement observed with monotherapy when using the highest dose of $\alpha$-lipoic acid (0.5% in the diet) or fidarestat (15 mg/kg BW). Reports of synergistic interactions in the prevention of diabetes-induced slowing of MNCV and reduction of EBF between $\alpha$-lipoic acid and $\gamma$-linolenic acid have previously appeared [8, 53]. These investigations have reported that treating diabetic rats with a combination of an aldose reductase inhibitor and angiotensin-converting enzyme inhibitor has synergistic effects on the neurovascular [54]. It has been reported that both MNCV and EBF were normalized by this combination therapy [54]. In regard to vascular reactivity impaired by diabetes, Keegan and colleagues have demonstrated that relaxation responses to acetylcholine in the corpus cavernosum were reduced by about 40% by diabetes [53]. Treating diabetic rats with either $\alpha$-lipoic acid or $\gamma$-linolenic acid alone did not significantly improve this deficit. However, when combination therapy was used, the endothelium-dependent relaxation was fully corrected. The authors concluded that $\alpha$-lipoic acid and $\gamma$-linolenic acid interact synergistically to improve nitric oxide–mediated neurogenic and endothelium-dependent relaxation of corpus cavernosum in experiment diabetes [53].

In summary, diabetic neuropathy is a multifactorial disorder and combination therapy may be the best approach in preventing vascular and neural dysfunction. These studies provide evidence that the combination of $\alpha$-lipoic acid and fidarestat provide an effective approach for treatment of diabetic vascular and neural dysfunction, and that fidarestat may promote the formation of dihydrolipoic acid in the diabetic state, contributing to a more effective treatment for impaired vascular relaxation in diabetes.

### REFERENCES

[1] Tomlinson, D. (1998) Future prevention and treatment of diabetic neuropathy. *Diabetes Metab.,* **24,** 79–83.

[2] Akbari, C. M., and LoGerfo, F. W. (1999) Diabetes and peripheral vascular disease. *J. Vasc. Surg.,* **30,** 373–384.

[3] Baynes, J. W., and Thorpe, S. R. (1999) Role of oxidative stress in diabetic complications: A new perspective on an old paradigm. *Diabetes,* **48,** 1–9.

[4] De Vriese, A. S., Verbeuren, T. J., Van de Voorde, J., Lameire, N. H., and Vanhoutte, P. M. (2000) Endothelial dysfunction in diabetes. *Br. J. Pharmacol.,* **130,** 963–974.

[5] Pieper, G. M., Langenstroer, P., and Gross, G. J. (1993) Hydroxyl radicals mediate injury to endothelium-dependent relaxation in diabetic rat. *Mol. Cell. Biochem.,* **122,** 139–145.

[6] Cameron, N. E., and Cotter, M. A. (1995) Neurovascular dysfunction in diabetic rats: Potential contribution of autoxidation and free radicals examined using transition metal chelating agents. *J. Clin. Invest.,* **96,** 1159–1163.

[7] Pieper, G. M., and Siebeneich, W. (1997) Diabetes-induced endothelial dysfunction is prevented by long-term treatment with the modified iron chelator, hydroxyethyl starch conjugated-deferoxamine. *J. Cardiovasc. Pharmacol.,* **30,** 734–738.

[8] Cameron, N. E., Cotter, M. A., Horrobin, D. H., and Tritschler, H. J. (1998) Effects of $\alpha$-lipoic acid on neurovascular function in diabetic rats: Interaction with essential fatty acids. *Diabetologia,* **41,** 390–399.

[9] Pieper, G. M., and Siebeneich, W. (1998) Oral administration of the antioxidant *N*-acetylcysteine, abrogates diabetes-induced endothelial dysfunction. *J. Cardiovasc. Pharmacol.,* **32,** 101–105.

[10] Keegan, A., Cotter, M. A., and Cameron, N. E. (1999) Effects of diabetes and treatment with the antioxidant $\alpha$-lipoic acid on endothelial and neurogenic responses of corpus cavernosum in rats. *Diabetologia,* **42,** 343–350.

[11] Obrosova, I. G., Fathallah, L., and Greene, D. A. (2000) Early changes in lipid peroxidation and antioxidative defense in diabetic rat retina: Effect of DL-$\alpha$-lipoic acid. *Eur. J. Pharmacol.,* **398,** 139–146.

[12] Gocmen, C., Secilmis, A., Kumcu, E. K., Ertug, P. U., Onder, S., Dikmen, A., and Baysal, F. (2000) Effects of vitamin E and sodium selenate on neurogenic and endothelial relaxation of corpus cavernosum in the diabetic mouse. *Eur. J. Pharmaccol.,* **398,** 93–98.

[13] Cameron, N. E., and Cotter, M. A. (1999) Effects of antioxidants on nerve and vascular dysfunction in experimental diabetes. *Diabetes Res. Clin. Pract.* **45,** 137–146.

[14] Cameron, N. E., Cotter, M. A., and Maxfield, E. K. (1993) Anti-oxidant treatment prevents the development of peripheral nerve dysfunction in streptozotocin-diabetic rats. *Diabetologia,* **36,** 299–304.

[15] Cameron, N. E., Cotter, M. A., Archibald, V., Dines, K. C., and Maxfield, E. K. (1994) Anti-oxidant and pro-oxidant effects on nerve conduction velocity, endoneurial blood flow and oxygen tension in non-diabetic and streptozotocin-diabetic rats. *Diabetologia,* **37,** 449–459.

[16] Karasu, C., Dewhurst, M., Stevens, E. J., and Tomlinson, D. R. (1995) Effects of anti-oxidant treatment on sciatic nerve dysfunction in streptozotocin-diabetic rats; comparison with essential fatty acids. *Diabetologia,* **38,** 129–134.

[17] Finegold, D., Lattimer, S. A., Nolle, S., Bernstein, M., and Greene, D.A. (1983) Polyol pathway activity and *myo*-inositol metabolism. *Diabetes,* **32,** 988–992.

[18] Mayer, J. H., and Tomlinson, D. R. (1983) Prevention of defects of axonal transport and nerve conduction velocity by oral administration of *myo*-inositol or an aldose reductase inhibitor in streptozotocin-diabetic rats. *Diabetologia,* **25,** 433–438.

[19] Tomlinson, D. R., Moriaity, R. J., and Heidi Mayer, J. (1984) Prevention and reversal of defective axonal transport and motor nerve conduction velocity in rats with experimental diabetes by treatment with the aldose reductase inhibitor sorbinil. *Diabetes,* **33,** 470–476.

[20] Kikkawa, R., Hatanaka, I., Yasuda, H., Kobayashi, N., and Shigeta, Y. (1984) Prevention of peripheral nerve dysfunction by an aldose reductase inhibitor in streptozotocin-diabetic rats. *Metabolism,* **33,** 212–214.

[21] Sima, A. A. F., Prashar, A., Zhang, W.-X., Chakrabarti, S., and Greene, D. A. (1990) Preventive effect of long-term aldose reductase inhibition (Ponalrestat) on nerve conduction and sural nerve structure in the spontaneously diabetic bio-breeding rat. *J. Clin. Invest.,* **85,** 1410–1420.

[22] Cameron, N. E., Cotter, M. A., Dines, K. C., Maxfield, E. K., Carey, F., and Mirrlees, D. J. (1994) Aldose reductase inhibition, nerve perfusion, oxygenation and function in streptozotocin-diabetic rats: Dose-response considerations and independence from a *myo*-inositol mechanism. *Diabetologia,* **37,** 651–663.

[23] Hirata, Y., Fujimori, S., and Okada, K. (1988) Effect of a new aldose reductase inhibitor, 8′-chloro-2′,3′-dihydrospiro-[pyrrolidine-3,6′(5′H)-pyrrolol[1,2,3-de][1,4]benzoxamine]-2, 5,5′-trione (ADN-138), on delayed motor nerve conduction velocity in streptozotocin-diabetic rats. *Metabolism,* **37,** 159–163.

[24] Yoshida, T., Nishioka, H., Yoshioka, K., Nakano, K., Kondo, M., and Terashima, H. (1987) Effect of aldose reductase inhibitor ONO 2235 on reduced sympathetic nervous system nerve disorders in STZ-induced diabetic rats. *Diabetes,* **36,** 6–13.

[25] Betteridge, D. J. (2000) What is oxidative stress? *Metabolism,* **49,** 3–8.

[26] Shackelford, R. E., Kaufmann, W. K., and Paules, R. S. (2000) Oxidative stress and cell cycle checkpoint function. *Free Radic. Biol. Med.,* **28,** 1387–1404.

[27] Sagara, Y., Dargusch, R., Chambers, D., Davis, J., Schubert, D., and Maher, P. (1998) Cellular mechanisms of resistance to chronic oxidative stress. *Free Radic. Biol. Med.,* **24,** 1375–1389.

[28] Terata, K., Coppey, L. J., Davidson, E. P., Dunlap, J. A., Gutterman, D. D., and Yorek, M. A. (1999) Acetylcholine-induced arteriolar dilation is reduced in streptozotocin-induced diabetic rats with motor nerve dysfunction. *Br. J. Pharmacol.,* **128,** 837–843.

[29] Coppey, L. J., Davidson, E. P., Dunlap, J. A., Lund, D. D., and Yorek, M. A. (2000) Slowing of motor nerve conduction velocity in streptozotocin-induced diabetic rats is preceded by impaired vasodilation in arterioles that overlie the sciatic nerve. *Int. J. Exp. Diabetes Res.,* **1,** 131–143.

[30] Coppey, L. J., Gellett, J. S., Davidson, E. P., Dunlap, J. A., Lund, D. D., and Yorek, M. A. (2001) Effect of antioxidant treatment of streptozotocin-induced diabetic rats on endoneurial blood flow, motor nerve conduction velocity and vascular reactivity of epineurial arterioles of the sciatic nerve. *Diabetes,* **50,** 1927–1937.

[31] Otter, D. J., and Chess-Williams, R. (1994) The effects of aldose reductase inhibition with ponalrestat on changes in vascular function in streptozotocin diabetes rats. *Br. J. Pharmacol.,* **113,** 576–580.

[32] Wakasugi, M., Noguchi, T., Inoue, M., Tawata, M., Shindo, H., and Onaya, T. (1991) Effects of aldose reductase inhibitors on prostacyclin ($PGI_2$) synthesis by aortic rings from rats with streptozotocin-induced diabetes. *Prostaglandins Leukot. Essent. Fatty Acids,* **44,** 233–236.

[33] Cameron, N. E., and Cotter, M. A. (1992) Impaired contraction and relaxation in aorta from streptozotocin-diabetic rats: Role of polyol pathway. *Diabetologia,* **35,** 1011–1019.

[34] Keegan, A., Jack, A.M., Cotter, M.A., and Cameron, N.E. (2000) Effects of aldose reductase inhibition on responses of the corpus cavernosum and mesenteric vascular bed of diabetic rats. *J. Cardiovasc. Pharmacol.,* **35,** 606–613.

[35] Coleman, M. D., Eason, R. C., and Bailey, C. J. (2001) The therapeutic use of lipoic acid in diabetes: A current perspective. *Environ. Toxicol. Pharmacol.,* **10,** 167–172.

[36] Packer, L., Kraemer, K., and Rimbach, G. (2001) Molecular aspects of lipoic acid in the prevention of diabetes complications. *Nutrition,* **17,** 888–895.

[37] Dincer, Y., Telci, A., Kayah, R., Yilmaz, I.A., Cakatay, U., and Akcay, T. (2002) Effect of $\alpha$-lipoic acid on lipid peroxidation and anti-oxidant enzyme activities in diabetic rats. *Clin. Exp. Pharmacol. Physiol.,* **29,** 281–284.

[38] Jones, W., Li, X., Zhi-Chao, Q., Perriott, L., Whitesell, R. R., and May, J. M. (2002) Uptake, recycling, and antioxidant actions of $\alpha$-lipoic acid in endothelial cells. *Free Radic. Biol. Med.,* **33,** 83–93.

[39] Haramaki, N., Han, D., Handelman, G. J., Tritschler, H. J., and Packer, L. (1997) Cytosolic and mitochondrial systems for NADH- and NADPH-dependent reduction of alpha-lipoic acid. *Free Radic. Biol. Med.,* **22,** 535–542.

[40] Bravenboer, B., Kappelle, A. C., Hamers, F. P. T., van Buren, T., Erkelens, D. W., and Gispen, W. H. (1992) Potential use of glutathione for the prevention and treatment of diabetic neuropathy in the streptozotocin-induced diabetic rat. *Diabetologia,* **35,** 813–817.

[41] van Dam, P. S. (2002) Oxidative stress and diabetic neuropathy: Pathophysiological mechanisms and treatment perspectives. *Diabetes Metab. Res. Rev.,* **18,** 176–184.

[42] Young, W. (1980) $H_2$ clearance measurement of blood flow: A review of technique and polarographic principles. *Stroke,* **11,** 552–564.

[43] Lou, M. F., Dickerson, J. E., Jr., Garadi, R., and York, B. M. Jr. (1988) Glutathione depletion in the lens of galactosemic and diabetic rats. *Exp. Eye Res.,* **46,** 517–530.

[44] Mihara, M., Uchiyama, M., and Fukuzama, K. (1980) Thiobarbituric acid value of fresh homogenate of rat as a parameter of lipid peroxidation in aging, CC14 intoxication, and vitamin E deficiency. *Biochem. Med.* **23,** 302–311.

[45] Siman, C. M., and Eriksson, U. J. (1997) Vitamin C supplementation of the maternal diet reduces the rate of malformation in the offspring of diabetic rats. *Diabetologia,* **40,** 1416–1424.

[46] Teichert, J., and Priess, R. (2002) High-performance liquid chromatographic assay for $\alpha$-lipoic acid and five of its metabolites in human plasma and urine. *J. Chromatog.,* **769,** 269–281.

[47] Coppey, L. J., Gellett, J. S., Davidson, E. P., Dunlap, J. A., Lund, D. D., and Yorek, M. A. (2002) Effect of treating streptozotocin-induced diabetic rats with sorbinil, *myo*-inositol or aminoguanidine on endoneurial blood flow, motor nerve conduction velocity and vascular function of epineurial arterioles of the sciatic nerve. *Int. J. Exp. Diabetes Res.,* **3,** 21–36.

[48] Arner, E. S., Nordberg, J., and Holmgren, A. (1996) Efficient reduction of lipoamide and lipoic acid by mammalian thioredoxin reductase. *Biochem. Biophysical Res. Commun.,* **225,** 268–274.

[49] Constantinescu, A., Pick, U., Handelman, G. J., Haramaki, N., Han, D., Podda, M., Tritschler, H. J., and Packer, L. (1995) Reduction and transport of lipoic acid by human erythrocytes. *Biochem. Pharmacol.,* **50,** 253–261.

[50] Williamson, J. R., Chang, K., Frangos, M., Hasan, K. S., Ido, Y., Kawamura, T., Nyengaard, J. R., van den Enden, M., Kilo, C., and Tilton, R. G. (1993) Hyperglycemic pseudohypoxia and diabetic complications. *Diabetes,* **42,** 801–813.

[51] Nakamura, J., Hamada, Y., Chaya, S., Nakashima, E., Naruse, K., Kato, K., Yasuda, Y., Kamiya, H., Sakakibara, F., Koh, N., and Hotta, N. (2002) Transition metals and polyol pathway in the development of diabetic neuropathy in rats. *Diabetes Metab. Res. Rev.*, **18,** 395–402.

[52] Stevens, M. J., Obrosova, I., Cao, X., Van Huysen, C., and Greene, D. A. (2000) Effects of DL-$\alpha$-lipoic acid on peripheral nerve conduction, blood flow, energy metabolism, and oxidative stress in experimental diabetic neuropathy. *Diabetes,* **49,** 1006–1015.

[53] Keegan, A., Cotter, M. A., and Cameron, N. E. (2001) Corpus cavernosum dysfunction in diabetic rats: Effects of combined alpha-lipoic acid and gamma-linolenic acid treatment. *Diabetes Metab. Res. Rev.*, **17,** 380–386.

[54] Cotter, M. A., Mirrlees, D. J., and Cameron, N. E. (2001) Neurovascular interactions between aldose reductase and angiotensin-converting enzyme inhibition in diabetic rats. *Eur. J. Pharmacol.*, **417,** 223–230.

